# Short experimental heatwaves have sublethal impacts on male reproduction in a model insect

**DOI:** 10.1242/jeb.250555

**Published:** 2025-08-01

**Authors:** Benjamin Cole, Ramakrishnan Vasudeva, Kay Dragoi, Josie Hibble, James King, Alexei A. Maklakov, Tracey Chapman, Matthew J. G. Gage

**Affiliations:** ^1^ School of Biological Sciences, University of East Anglia, Norwich, NR4 7TJ, UK; ^2^ Faculty of Environment, Science and Economy, Ecology and Conservation, University of Exeter, Penryn, Cornwall, TR10 9FE, UK

**Keywords:** Climate change, Heatwaves, Insect fertility, Male susceptibility, *Tribolium*

## Abstract

Heatwaves are becoming more common and severe. Previous work has highlighted male insects as being particularly vulnerable to multi-day continuous heatwaves, yet our understanding of short duration heatwave impacts on insects is limited. Here, we assessed the impacts of short, simulated heatwave exposures (2, 5 and 10 h) using ecologically relevant temperatures (42, 44, 46, 48 and 50°C) on survival, reproductive output, testes volume and sperm length in *Tribolium castaneum*. We show that reproductive output is compromised at lower temperatures than survival, especially during the shortest heatwaves, supporting the notion that thermal fertility limits are lower than thermal viability limits. Furthermore, testes volume was reduced by 40% after a 10 h exposure at 42°C and sperm length decreased by 2.7% after an exposure of 42°C for just 2 h. This highlights that even short heat exposure can impact male fertility and reproductive trait morphology at temperatures below viability limits.

## Teaser

**Summary:** Heatwaves are becoming more common and severe, posing a threat to organisms. Short, simulated heatwaves affect male insects, and reproduction is compromised at sublethal temperatures.

## INTRODUCTION

Significant changes to Earth's climate are being seen due to human activities, with mean global temperature predicted to reach up to 1.9°C higher than pre-industrial times by 2028 (World Meteorological Organisation, 2024). In addition to increases in mean global temperature, incidences of extreme heat are also rising. Heatwaves are becoming broadly more common and intense, including in regions where they were previously rare (Meehl and Tebaldi, 2004; Perkins-Kirkpatrick and Lewis, 2020). In particular, short duration heatwaves and their impacts on ecosystems and the environment are expected to increase in frequency (Trenberth, 2018). Understanding the ecological and evolutionary implications of these heatwaves is critical as climatic extremes are shifting more rapidly than mean temperature (Seneviratne et al., 2021).

With anthropogenic climate change occurring at accelerating rates in all geographical regions (World Meteorological Organisation, 2024), there is emerging evidence that it has caused species extinctions (Ceballos et al., 2015), with insects highlighted as being particularly at risk (Dunn, 2005; Wiens, 2016; Wagner et al., 2021). Understanding the mechanisms underpinning current and future insect declines under climate change is becoming an increasingly important research focus, with extreme heat often linked to decreasing insect survival (Ma et al., 2020). However, sub-lethal impacts of heat on biological functions are less well understood. Recent research has shown that thermal fertility limits (TFLs) are reached at lower temperatures than critical thermal limits (CTLs) in some species (Parratt et al., 2021). Furthermore, male TFLs have been shown to predict species distribution and extinction risk better than CTLs (Parratt et al., 2021; van Heerwaarden and Sgrò, 2021).

Many studies have assessed the effect of extreme heat on multiple aspects of male insect reproduction including spermatogenesis (Canal Domenech and Fricke, 2023), sperm motility, mobility and morphology (Uy et al., 2015; Porcelli et al., 2016; Iossa et al., 2019), sperm number (Vasudeva et al., 2014; Sales et al., 2018), sperm viability (Sales et al., 2018; Martinet et al., 2020; Campion et al., 2023), sperm storage (Sales et al., 2024), testes volume (Vasudeva et al., 2014; Sales et al., 2021), courtship and mating behaviour (Grandela et al., 2023; Ratz et al., 2024) and parental care (Pilakouta et al., 2023). These studies have highlighted the range of fertility traits that can be affected. However, research on the impact of short-term heat remains limited (Dougherty et al., 2024), which is a particular concern given that shorter and more intense heatwaves are increasing in frequency (Trenberth, 2018).

Using *Tribolium castaneum*, a widely used model in evolution and ecology (Pointer et al., 2021; Campbell et al., 2022), Sales et al. (2018) showed that subjecting virgin males, but not virgin females, to a simulated heatwave before mating severely reduced reproductive output. Subjecting males to heatwave conditions of 42°C for 5 continuous days halved male fertility, with a further heatwave sterilising most individuals, which was linked to decreased sperm viability and number. Such long-term heatwaves were also shown to reduce testes volume by half, which recovered to control levels ∼25 days post-heatwave exposure (Sales et al., 2021). Under future climate projections, ‘extremely hot days’ with temperatures over 50°C are expected to become increasingly common within areas of *T. castaneum*’s global distribution (Campbell et al., 2022; Christidis et al., 2023). It is important to understand the consequences of such intense conditions under ecologically relevant scenarios, as peak temperatures within a heatwave may only be experienced for a few hours in a single day (Holmes et al., 2015; Oliveira et al., 2021). Therefore, there is a pressing need to investigate how exposure to various short duration heatwave conditions may impact male survival and fertility and understand the potential implications this may have on insect populations.

This has been further emphasised by recent studies applying the thermal death time (TDT) framework, which integrates both the duration and intensity of thermal exposures to predict biological damage. While initially focused on describing survival thresholds, such approaches are increasingly being used to understand sublethal impacts on fertility, highlighting that different traits may have distinct thermal sensitivities and damage accumulation rates (Rezende et al., 2014; Jørgensen et al., 2019; Ørsted et al., 2024). Our study contributes to a growing understanding of such interacting factors by testing the impacts of ecologically relevant simulated heatwaves with varying duration (2, 5 or 10 h) and intensity (42, 44, 46, 48 or 50°C) on male survival, reproductive output, testes volume and sperm length using *T. castaneum.* We predict that fertility and associated traits could be more sensitive than survival in general.

## MATERIALS AND METHODS

### Line maintenance and experimental individuals

*Tribolium castaneum* (Herbst 1797) beetles used in these experiments were from the Krakow Super Strain (KSS) outbred stock line (details on the set-up are given in Dickinson, 2018). Stock populations were maintained in 12×12×12 cm plastic tubs half-filled with fodder (a 9:1 volume ratio of organic flour and yeast) and topped with an even layer of oats for traction. Populations were kept at constant standard conditions of 30±1°C and 60±10% relative humidity (RH) under a 16 h:8 h light:dark photoperiod. Experimental individuals were obtained as the offspring of ∼300 mature adults selected at random from the stock line. These individuals were allowed to mate randomly and oviposit for 7 days in a fresh tub before the removal of adults by mechanical sieving, leaving only the fodder with oviposited eggs in this new population.

Individuals were sexed at the pupal stage by visual identification of sexually dimorphic genital papillae (on day 18 of development) and then kept in single-sex groups of 20 individuals for a further 10 days to allow for development to sexual maturity. Groups were kept in 6 cm Petri dishes filled with 3 g of fodder and topped with an even layer of oats. Once matured, females were identified with a dot of Uni Posca non-toxic marker (Uni-ball, Tokyo, Japan) on the dorsal thorax.

### Experimental heatwave treatments

Heatwave conditions were applied using either an A.B. Newlife 75 Mk4 forced air egg incubator or an A.B. Newlife 75 Mk 4 moving air incubator (A.B. Incubators, Suffolk, UK). These conditions were selected based on previous research, which established the reproductive optimum of males (see Sales et al., 2018). Virgin male beetles were exposed to either heatwave conditions (42, 44, 46, 48 or 50°C ±1°C, 60±10% RH) or control conditions (30±1°C, 60±10% RH) for 2, 5 or 10 h in single-sex groups of 20 individuals in 6 cm Petri dishes filled with 3 g of fodder and a layer of oats. Temperature was checked every 30 min using a digital thermometer integrated into the incubator and an additional mercury thermometer placed in the incubator. No recorded temperature was above or below 1°C from the set point.

### Experimental protocol

#### Survival and fertility

Virgin, sexually mature males (48–72 h post-eclosion) were subject to heatwave conditions with controls run in parallel (conditions described above). All individuals then experienced a further rest period at 30±1°C for 24 h. Females were sourced from the same population but were maintained constantly under standard conditions in identical density. Individual males were visually assessed at this time, and survivors were paired with an age-matched virgin female for 48 h at 30±1°C in a 7 ml mating vial. Sample sizes for survival and reproductive output assays are summarised in Table 1. These mating vials were filled with ∼2.4 g of fodder and topped with a few pieces of oats for traction. These mating vials were retained to ensure successful ejaculate transfer, assessed by the visual presence of larval tracks/offspring.

**Table 1.** Number of *Tribolium castaneum* individuals subject to each heatwave temperature/duration condition for the survival assay and number of survivors that subsequently went through the reproductive output assay

	Temperature (°C)	Sample size		
		2 h	5 h	10 h
Survival	30	58	60	60
	42	40	40	60
	44	35	38	60
	46	34	40	60
	48	70	69	60
	50	70	61	60
Reproductive output	30	53	54	51
	42	38	37	56
	44	28	32	60
	46	32	32	59
	48	65	65	32
	50	68	52	0*

*No individuals were assayed because of 100% mortality during the heatwave exposure.

Females from the above mating assays were then moved into individual 6 cm Petri dishes (filled with 3 g of fodder and topped with an even layer of oats) and allowed to oviposit. Oviposition occurred across two separate 10 day blocks (20 days in total) to reduce cannibalism associated with overlapping offspring developmental life stages (Park et al., 1965). After 20 days, the females were removed, and the oviposited eggs were allowed to develop until maturity for an additional 35 days under standard developmental conditions (30±1°C, 60±10% RH). The reproductive output of each pair was then assessed as the number of mature adults produced from 20 days of oviposition (equating to ∼51% of a female's lifetime reproductive output; see p. 31, Dickinson, 2018).

#### Testes and sperm measurements

An additional cohort of males that experienced identical experimental conditions were allocated to quantifying testes volume and total sperm length. These individuals were frozen at −20°C immediately after being subject to either heatwave or control conditions (as described above). All samples were blinded to the user by a random code at this point to avoid any unconscious bias during morphological measurements. Measurements were taken from individuals that survived the heatwaves for all experimental groups apart from those subject to 50°C for 10 h, where all males had died during the exposure period. Testes dissections and measurements were carried out as described in Sales et al. (2021). The testes of 10 males were measured per duration/heatwave condition. Total sperm length measurements were taken as described in Godwin et al. (2017) and Vasudeva et al. (2019). Twenty individual mature intact sperm were measured per male (*N*=10 males per duration/heatwave condition).

### Statistical analysis

All data were analysed using R version 4.4.1 (http://www.R-project.org/) in RStudio Version 2024.04.2+764 (https://posit.co). Plots were created using ‘ggplot2’ (Wickham, 2016). Data manipulation was done using ‘tidyverse’ (Wickham et al., 2019). The impact of heatwave temperature on survival for each of the three heatwave durations was initially assessed using a binomial GLM. Firth's penalised logistic regression from the brglm2 package was used where near-separation or complete separation issues caused model convergence issues (https://CRAN.R-project.org/package=brglm2). Fit of the models was then assessed using ‘performance’ (Lüdecke et al., 2021) and residuals were visually evaluated.

Reproductive output data were first censored where individuals escaped from or died during the reproductive output assay (*N*=31). Zero-inflated negative binomial generalised linear models (GLMs) (Zeileis et al., 2008) were used to analyse data after assessing models for over- or under-dispersion and comparing goodness of fit using ‘performance’ and ‘lmtest’ (Zeileis and Hothorn, 2002).

Testes volume data were analysed using Gaussian GLMs and models, and residuals were tested. Where residuals showed heteroscedasticity, gamma GLMs were employed, and models were compared for goodness of fit. Average sperm length data, grouped by heatwave condition, were analysed using gamma GLMs and models and residuals were tested using ‘performance’ and ‘lmtest’.

## RESULTS AND DISCUSSION

Our findings show that reproductive traits were impacted at sublethal temperatures when male *T. castaneum* beetles were exposed to heatwave conditions from 42°C to 50°C for 2, 5 and 10 h. Some reproductive traits were also more thermally sensitive than others. For example, sperm length showed reductions at lower temperatures than reproductive output for all heatwave durations. Our results are in line with existing findings across taxa, which report sensitivity to heat in the reproductive traits of males (e.g. Canal Domenech and Fricke, 2023; Grandela et al., 2023; Meena et al., 2024; Ratz et al., 2024). Statistics are summarised in Table 2.

**Table 2.** Summary statistics for survival, reproductive output, testes volume and sperm length data under various heatwave conditions

Treatment	Survival			20 day reproductive output			Testes volume (mm^3^)			Sperm length (μm)		
	Survivors (*n*/*N*)	*z*	*P*	Mean±s.e.m.	*z*	*P*	Mean±s.e.m.	*t*	*P*	Mean±s.e.m.	*t*	*P*
2 h exposure												
30°C	54/58	–	–	207±15.3	–	–	0.260±0.014	–	–	86.4±0.758	–	–
42°C	38/40	0.291	0.771	218±19.8	1.058	0.290	0.299±0.018	1.579	0.120	84.2±0.640	−2.099	0.041
44°C	35/35	1.158	0.247	183±15.9	−1.843	0.065	0.210±0.021	−1.969	0.054	81.5±0.968	−4.560	<0.001
46°C	32/34	0.085	0.932	186±16.7	−0.908	0.364	0.224±0.023	−1.427	0.159	81.8±0.821	−4.346	<0.001
48°C	65/70	−0.025	0.980	217±12.4	−0.230	0.818	0.225±0.012	−1.375	0.175	81.1±0.623	−4.977	<0.001
50°C	69/70	1.387	0.165	139±8.7	−4.302	<0.001	0.184±0.017	−3.018	0.004	80.7±0.623	−0.5352	<0.001
5 h exposure												
30°C	59/60	–	–	262±10.7	–	–	0.273±0.011	–	–	88.1±0.577	–	–
42°C	39/40	−0.290	0.772	222±19.6	−0.536	0.592	0.254±0.019	−0.764	0.448	83.6±0.686	−4.367	<0.001
44°C	35/38	−1.381	0.167	217±11.6	−1.541	0.123	0.226±0.017	−1.881	0.065	81.4±0.727	−6.581	<0.001
46°C	34/40	−2.128	0.033	190±20.9	−1.450	0.147	0.245±0.020	−1.097	0.278	82.8±0.763	−5.162	<0.001
48°C	67/69	−0.457	0.647	201±12.6	−1.985	0.047	0.201±0.018	−2.847	0.006	79.8±0.668	−8.344	<0.001
50°C	54/61	−1.875	0.061	120±12.5	−6.111	<0.001	0.176±0.021	−3.865	<0.001	81.8±0.740	−6.267	<0.001
10 h exposure												
30°C	56/60	–	–	264±10.7	–	–	0.292±0.010	–	–	84.4±0.587	–	–
42°C	57/60	0.362	0.717	255±12.8	0.292	0.770	0.174±0.009	4.471	<0.001	82.5±0.711	−1.836	0.072
44°C	60/60	1.496	0.135	235±12.3	−0.840	0.401	0.183±0.007	4.077	<0.001	80.1±0.839	−4.228	<0.001
46°C	59/60	1.187	0.235	219±10.2	−2.026	0.043	0.205±0.020	3.147	0.003	81.5±0.915	−2.836	0.006
48°C	32/60	−4.302	<0.001	90±19.4	−2.837	0.005	0.155±0.010	5.356	<0.001	80.5±0.668	−3.824	<0.001
50°C	0/60	−4.837	<0.001	–	–	–	0.086±0.011	8.754	<0.001	81.7±0.524	−2.684	0.010

Statistics represent a comparison between each group and their respective control.

**Fig. 1.**
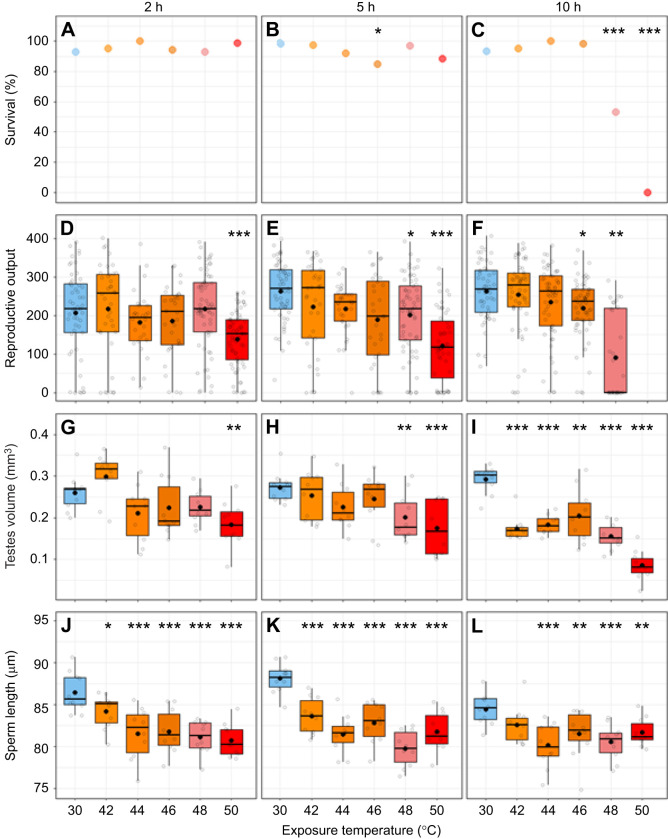
**Effects of varying heatwave temperature/duration conditions on male *Tribolium castaneum*.** (A–C) Survival, (D–F) 20 day reproductive output, (G–I) testes volume and (J–L) sperm length following exposure to 30, 42, 44, 46, 48 and 50°C for 2, 5 and 10 h. For D–I, raw data points are plotted as open jittered circles. For J–L, the mean sperm length per male is plotted as open jittered circles. Boxplots contain a median line, mean dot and interquartile range box. Significance values representing a comparison between 30°C and the experimental heatwave condition are denoted by asterisks: *P<0.05, **P<0.01, ***P<0.001. Sample size for survival from left to right for each exposure duration: 2 h (A): 58, 40, 35, 34, 70, 70; 5 h (B): 60, 40, 38, 40, 69, 61; 10 h (C): 60, 60, 60, 60, 60, 60. Sample size for reproductive output for each exposure duration: 2 h (D): 53, 38, 28, 32, 65, 68; 5 h (E): 54, 37, 32, 32, 65, 52; 10 h (F): 51, 56, 60, 59, 32 (all individuals exposed to 50°C for 10 h died before the reproductive assay). Sample size for testes volume was 10 individuals per group. The sample size for sperm length was 20 individual sperm from 10 males per group.

The survival of males exposed to heatwave conditions for 2 or 5 h was high across all groups, with no significant reduction compared with controls, except for a 5 h exposure at 46°C. However, this difference was not observed at higher temperatures. Survival started to drop dramatically compared with controls in males exposed to higher temperatures for 10 h (Fig. 1A–C). Males exposed to heatwave conditions of 48°C and 50°C for 10 h showed a marked reduction in survival. At 48°C, only 53% of males survived, and at 50°C, none survived (compared with 93.3% in the control group). Here, we demonstrate that male *T. castaneum* survival was resilient to heatwave conditions until ∼11–13°C above their optimum at 35°C (Sales et al., 2018) before hitting an upper limit when exposed to very intense heat (48°C and 50°C for 10 h).

By contrast, male reproductive output and reproductive morphology were less resilient across a range of short duration heat exposures. As exposure duration and intensity increased, we observed increasingly negative impacts on reproductive output. We found that temperatures as low as 46°C caused a significant reduction in reproductive output after exposure to heatwave conditions for 10 h. At 48°C and 50°C, there were significant reductions following heat exposure of only 5 h and 2 h, respectively. Heatwave exposure at 50°C for 2 h reduced male reproductive output by 33% compared with controls (Fig. 1D). Heatwave exposure at 48°C and 50°C for 5 h reduced male reproductive output by 23% and 55%, respectively (Fig. 1E). Heatwave exposure at 46°C and 48°C for 10 h reduced male reproductive output by 17% and 66%, respectively (Fig. 1F). All individuals exposed to 50°C for 10 h died before the reproductive assay; therefore, no reproductive output data were collected.

The relationship between heatwave duration and intensity shown in this study further highlights the importance of considering the two factors simultaneously when assessing the impact of heat on insects. This is particularly relevant when considering future climate change scenarios where both short duration and more intense heatwaves are predicted to become more prevalent (Trenberth, 2018). Moreover, there is a need to consider trait-specific responses to combinations of these factors.

Consistent with previous studies (Sales et al., 2021; Hu et al., 2022), we found that testes volume was significantly impacted by heatwave exposure. This effect was observed following exposure to very brief heatwave conditions. Males exposed to heatwave conditions of 50°C for 2 h showed reduced testes volume by 29% compared with controls (Fig. 1G). For 5 h heatwave exposure, 48°C and 50°C reduced testes volume by 26% and 36%, respectively (Fig. 1H). For 10 h exposure, all heatwave temperatures reduced testes volume compared with controls, with even the lowest exposure temperature of 42°C resulting in a 40% drop in testes volume (Fig. 1I). This reduction in testes volume remained relatively consistent over 44°C (37%) and 46°C (30%), and further reductions were seen at 48°C (47%) and at 50°C, where testes volume was reduced by 70%. For both 2 h and 5 h heatwaves, the temperatures that resulted in significant reductions in testes volume were the same as those at which significant reductions in reproductive output occurred (50°C and 48°C, respectively). For 10 h exposures, testes volume was significantly reduced at a lower temperature (42°C) than that at which reproductive output was first compromised (46°C). Broadly, these findings suggest that reductions in testes volume are associated with reduced male reproductive output and that testes volume is highly thermally sensitive.

Heat-related damage to insect sperm has been documented previously (Martinet et al., 2020; Sales et al., 2021; Canal Domenech and Fricke, 2023; Lv et al., 2024). In *T. castaneum*, a 5 day, 42°C heatwave reduced sperm survival by ∼60% and sperm count by 75% (Sales et al., 2018). Here, we found that even the shortest and least intense heatwave exposures tested in this study caused a decrease in sperm length. All heatwave exposures for 2 h reduced sperm length compared with controls (Fig. 1J), with exposure at 42°C reducing sperm length by 2.7%. This reduction in total sperm length generally worsened with increasing temperature, seeing decreases at 44°C (5.7%), 46°C (5.4%), 48°C (6.2%) and 50°C (6.7%). Similarly, for both 5 h and 10 h exposures, we observed reduced sperm length compared with controls (Fig. 1K,L). For 5 h exposure, 42°C resulted in a 5.1% reduction, with further decreases at 44°C (7.6%), 46°C (6%), 48°C (9.5%) and 50°C (7.3%). For 10 h exposure, all heatwave temperatures, except 42°C, resulted in significantly reduced sperm length compared with controls. After 44°C exposure, there was a 5.1% reduction, with similar decreases at 46°C (3.5%), 48°C (4.7%) and 50°C (3.3%). It is unclear exactly what mechanism gives rise to these sperm length reductions, and future work on this will be insightful.

Spermatogenesis occurs throughout the adult life stage in *T. castaneum*, but the exact duration of spermatogenesis and spermiogenesis is unknown. However, in other insects, these processes occur over a number of days (e.g. 10 days for spermatogenesis and 5 days for spermiogenesis in *Drosophila melanogaster*: Rohmer, 2004; Fabian and Brill, 2012; and 12 days for spermatogenesis in *Haematobia irritans*: Basso et al., 2011). Considering the short duration between the initiation of heat exposure and freezing of samples in this study (2–10 h), we suggest that the mechanisms underpinning sperm length change in response to heat exposure may be linked to disruption of spermiogenesis in nearly mature sperm or through direct impacts on mature sperm. This is supported by an observed increase in sperm length variation within individuals, associated with increasing temperatures of heatwave conditions (see Fig. S1). Further work assessing the specific morphological changes associated with such sperm length variation may elucidate the mechanisms behind the impacts on sperm and male reproductive output observed in this study.

Broadly, we have highlighted that the thermal sensitivity of male reproductive traits is even greater than previously demonstrated (e.g. Sales et al., 2018, 2021). Our results showing trait-specific sensitivity also align with recent work on other taxa. For example, in *Drosophila suzukii*, traits (survival, coma induction and productivity) were shown to have different sensitivities to heat stress exposure, and the stress durations required to produce a 50% reduction in each trait often varied greatly (Ørsted et al., 2024). That study, utilising thermal dose–time models, builds on earlier work showing that the time–temperature relationship underlying thermal damage differs across traits (Jørgensen et al., 2019), and future work integrating such approaches would be insightful.

It would be interesting to expand future heatwave studies to test whether trait-specific variability changes after multiple days of exposure, with and without a rest phase. This may reveal the relative vulnerability of specific traits to natural heatwaves, whose intensity would vary over several days (Frich et al., 2002; Christidis et al., 2023). It is also unclear whether some traits can recover or harden after exposure to extreme conditions (e.g. 48°C and 50°C) under natural heatwave scenarios, as potentially compounding effects from repeated exposures may be modulated by periods of non-stressful conditions (e.g. benign cooler night-time and daytime temperatures), allowing time for physiological repair and fitness recovery (Bai et al., 2019). Previous work found that males can recover reproductive function after longer exposure to less intense experimental heatwave conditions (Sales et al., 2021). Therefore, it will also be important to understand whether there is a general recovery of reproductive potential in males exposed to shorter but more intense heatwave conditions as used in this study.

Our study focused on the effects of heatwave conditions that may be experienced by this species in its natural environment, and which are likely to be increasingly common in the future (Zittis et al., 2021; Campbell et al., 2022). We show that reproductive output is sensitive to sublethal short heatwaves. However, we recognise that the thermal homogeneity and potential lack of interacting factors in our study may have constrained strategies such as moving to more benign microhabitats (e.g. dropping behaviour in aphids; Ma and Ma, 2012), which may alleviate the impact of extreme heat (Terlau et al., 2023). In future studies, it will be necessary to explore these factors and assess whether different species can adapt to increasingly severe short-term heat exposures (Kellermann and van Heerwaarden, 2019) or whether they are likely to be overwhelmed by the transient and unpredictable nature of extreme thermal events (van de Pol et al., 2017).

## Supplementary Material

10.1242/jexbio.250555_sup1 Supplementary information
