# Supplementary material for: Short experimental heatwaves have sublethal impacts on male reproduction in a model insect
Source: J Exp Biol. 2025 Aug 1;228(15):jeb250555. doi: 10.1242/jeb.250555 (PMC12377809; doi:10.1242/jeb.250555)
Supplement: Supplementary information [file jexbio-228-250555-s1.pdf]

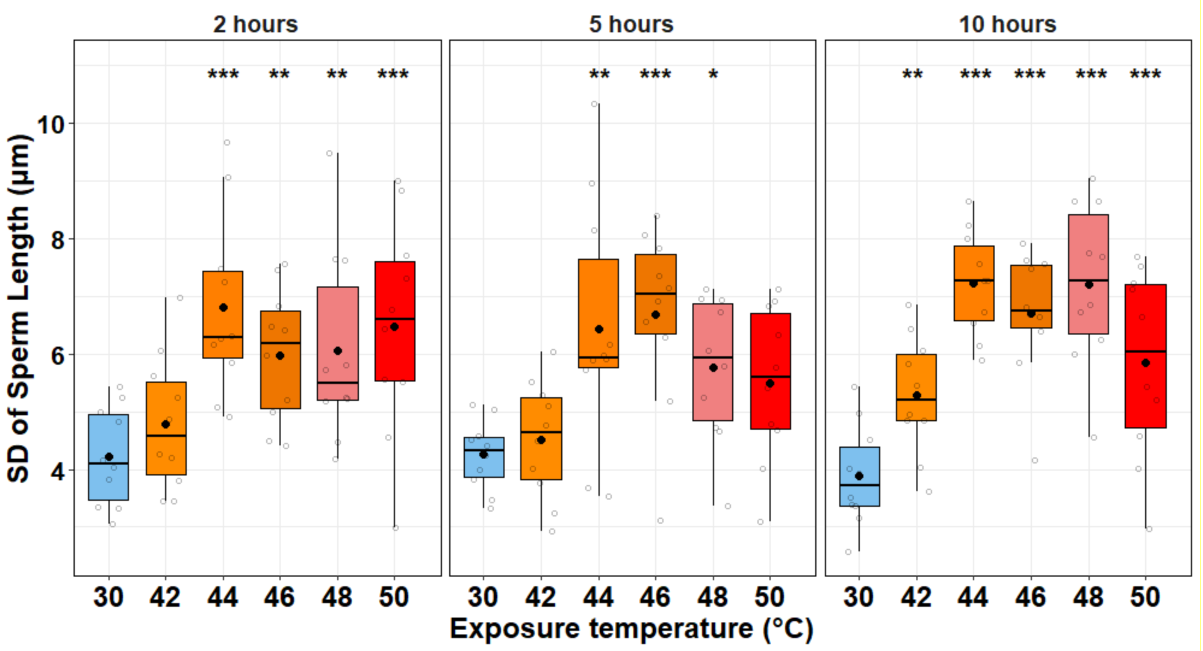

Fig. S1. Effects of varying heatwave conditions on the standard deviation of sperm length within males. The mean sperm length standard deviation per male is plotted as open jittered circles. Boxplots contain a median line, mean dot and interquartile range box. Significance values representing a comparison between 30°C and the experimental heatwave condition are denoted by stars: * = P <0.05, ** = P < 0.01, *** = P < 0.001. The sample size for sperm length variation consisted of 20 individual sperm from 10 males across all groups.

The standard deviation of sperm length for each male was calculated. Linear mixed models were fitted separately for each exposure duration with temperature as a fixed effect and Male ID as a random effect. Our results show a significant increase in sperm length variability at higher temperatures across all durations (Fig. S1). All heatwave exposures for 2 h increased the standard deviation of sperm length when compared to controls, except for 42°C ($t = 0.933$, $p = 0.356$). Variability increased by 61.1% at 44°C ($t = 4.234$, $p < 0.001$), 41.7% at 46°C ($t = 2.881$, $p = 0.006$), 43.3% at 48°C ($t = 3.008$, $p = 0.004$), and 53.1% at 50°C ($t = 3.683$, $p < 0.001$), relative to controls. In line with the 2 h exposure results, a 5 h exposure at 42°C did not significantly affect sperm length variation when compared to controls ($t = 0.415$, $p = 0.679$). In contrast, exposure to 44°C resulted in a 51.3% increase ($t = 3.459$, $p = 0.001$). Variability remained high at more extreme temperatures, with increases of 57.2% at 46°C ($t = 3.862$, $p < 0.001$), 35.3% at 48°C ($t = 2.390$, $p = 0.020$), and 29.3% at 50°C ($t = 1.974$, $p = 0.053$), although the latter only approached significance. Exposure to

Journal of Experimental Biology • Supplementary information

heatwave conditions for 10 h significantly increased sperm length variability at all temperatures tested. Variability increased by 36.0% at 42°C ($t = 2.792$, $p = 0.008$), with larger increases at higher temperatures: 85.5% at 44°C ($t = 6.637$, $p < 0.001$), 72.5% at 46°C ($t = 5.618$, $p < 0.001$), 85.3% at 48°C ($t = 6.614$, $p < 0.001$), and 49.9% at 50°C ($t = 3.883$, $p < 0.001$), relative to controls.

These findings demonstrate that prolonged heat exposure consistently elevates sperm length variability, highlighting a potential sensitivity of sperm morphology to heatwave conditions. This increase in variability may have a role in the observed reductions in male reproductive output.
